# Pair bonding and disruption impact lung transcriptome in monogamous Peromyscus californicus

**DOI:** 10.1186/s12864-023-09873-6

**Published:** 2023-12-19

**Authors:** A. Naderi, K. Liles, T. Burns, B. Chavez, K-T. Huynh-Dam, H. Kiaris

**Affiliations:** 1 Department of Drug Discovery and Biomedical Sciences, College of Pharmacy, University of South Carolina, Columbia, SC USA; 2 Department of Mathematics and Computer Sciences, Claflin University, Orangeburg, SC USA; 3 Department of Biology, Claflin University, Orangeburg, SC USA; 4 Peromyscus Genetic Stock Center, University of South Carolina, Columbia, SC USA

**Keywords:** Deer mice, Outbred, Transcriptome, Expression signature, Peromyscus, Monogamous, Cancer

## Abstract

Social interactions affect physiological and pathological processes, yet their direct impact in peripheral tissues remains elusive. Recently we showed that disruption of pair bonds in monogamous *Peromyscus californicus* promotes lung tumorigenesis, pointing to a direct effect of bonding status in the periphery (Naderi et al., 2021). Here we show that lung transcriptomes of tumor-free *Peromyscus* are altered in a manner that depends on pair bonding and superseding the impact of genetic relevance between siblings. Pathways affected involve response to hypoxia and heart development. These effects are consistent with the profile of the serum proteome of bonded and bond-disrupted *Peromyscus* and were extended to lung cancer cells cultured in vitro, with sera from animals that differ in bonding experiences. In this setting, the species’ origin of serum (deer mouse vs FBS) is the most potent discriminator of RNA expression profiles, followed by bonding status. By analyzing the transcriptomes of lung cancer cells exposed to deer mouse sera, an expression signature was developed that discriminates cells according to the history of social interactions and possesses prognostic significance when applied to primary human lung cancers. The results suggest that present and past social experiences modulate the expression profile of peripheral tissues such as the lungs, in a manner that impacts physiological processes and may affect disease outcomes. Furthermore, they show that besides the direct effects of the hormones that regulate bonding behavior, physiological changes influencing oxygen metabolism may contribute to the adverse effects of bond disruption.

**Supplementary Information:**

The online version contains supplementary material available at 10.1186/s12864-023-09873-6.

## Introduction

Both epidemiological and experiential evidence suggests that social interactions, especially between couples, modulate physiological processes and the outcome of various pathologies, including cancers. There have been both positive and negative effects depicted in the literature regarding marriage and widowhood. Males, in particular, have shown a greater sensitivity to death associated with widowhood than females [1–11].

It is estimated that less than 10% of mammals, with humans included, form pair bonds that are based on mating [12–14]. Laboratory mice (Mus musculus) are powerful in illuminating several physiological and pathological processes but are of limited value in modeling the effects of social interactions. Mice which do not develop long-term pair bonds, prohibit studies on the effects of pair bonds and their disruption under physiological conditions and in pathology [15]. Notwithstanding these limitations of laboratory mice, monogamous deer mice (genus Peromyscus) can model “widowhood” in the context of cancer, confirming that upon pair-bond disruption, males are more sensitive to lung tumorigenesis [15–18]. At least in part, the effects of “widowhood”, in this Peromyscus-based model are mediated by soluble factors that are present in the hosts’ sera and instruct the acquisition of bonding-associated expression profiles by the cancer cells [16]. It is plausible that such factors would also be operational under physiological conditions influencing gene expression, and therefore function, of peripheral tissues.

We tested these hypotheses by assessing the effects of bonding experiences in the transcriptome of monogamous *P. californicus* in the lungs. We also evaluated how the sera from bonded, virgin, and bond-disrupted deer mice influence the clustering of transcriptomes of lung cancer cells cultured in vitro and tested if the resulting gene signatures bear prognostic value.

## Results

### Clustering of lung transcriptomes according to bonding history

**Fig. 1:**
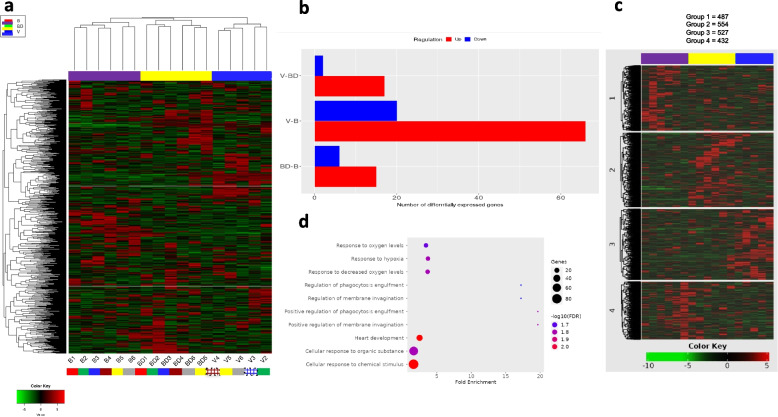
Expression profile of lung tissue from male P. californicus differing in bonding history. **a**. Unsupervised hierarchical clustering of whole transcriptome RNAseq data. Siblings are indicated by the same color coding. For example, B2, BD2, and V2 are siblings. Squared boxes indicate cousins. For example, B3 and BD3 are siblings and V3 is their cousin. **b.** Differential gene expression in whole transcriptome data. The number of differentially expressed genes is indicated. Gene IDs, fold change and adjusted P values are indicated in Supplementary Table 1. FDR cutoff: 0.1 and fold change ≥ 2. **c**. Among the four groups derived from K-means clustering, pathway enrichment analysis (GO analysis for biological processes) revealed consistent functions in group 1 (Supplementary Table 3). d. Bubble plot of top 10 enriched pathways. B, bonded; BD, bond-disrupted; V, Virgins. FPKM values were used, and the 1,000 most variable genes were considered

RNAseq was performed in the lungs of bonded (n-6), bond-disrupted (*n* = 6), and virgin (*n* = 5) male *P. californicus*. As shown in Fig. 1, unsupervised hierarchical clustering of RNA sequencing data indicated that animals were accurately discriminated according to their bonding status and past experiences. The fact that virgin animals are housed in groups of 2 or 3 animals and yet, they were not clustered together with the bonded animals, suggests that social interactions that are not based on mating (virgin group) do not substitute for pair bonding (bonded group). To assess the effects of genetic resemblance on gene clustering, the experiment involved siblings that were distributed into three different groups. (coded in the same color in Fig. 1a). Two mice from the virgin group were cousins to their corresponding mice in the other two groups (Fig. 1a). These animals did not cluster together, suggesting that the effects of genetic similarity in gene expression are masked by the effects of pair bonding and disruption, which induce more potent expression signatures. Thus, genetic variations between individuals produce less prominent effects than bonding history in the lung.

A series of differentially expressed transcripts were identified and are shown in Fig. 1b and Supplementary Table 1 to Fig. 1b. Adjusted P values and fold change in each pairwise comparison are indicated in Supplementary Table 1 to Fig. 1b. GO analysis for biological processes in the differentially expressed genes reveal significant enrichment of pathways involved in aminoacid metabolism, between bond-disrupted and virgin groups (Supplementary Table 2 to Fig. 1b). It is important however to interpret the result with caution since only one gen (Hibadh) was differentially expressed in all enrichment pathways. Then analysis of gene expression patterns using K-means clustering (k = 4) was performed for the 2,000 most variable genes to unveil clusters of coregulated genes. Among the four groups that developed (groups 1–4), a significant enrichment for biological processes was revealed only for the genes of group 1. (Fig. 1c). Among these processes, notable functions involved responses to hypoxia and heart development (Fig. 1d and Supplementary Table 3 to Fig. 1c).

### Clustering of cancer cells and bonding history of serum donors

**Fig. 2:**
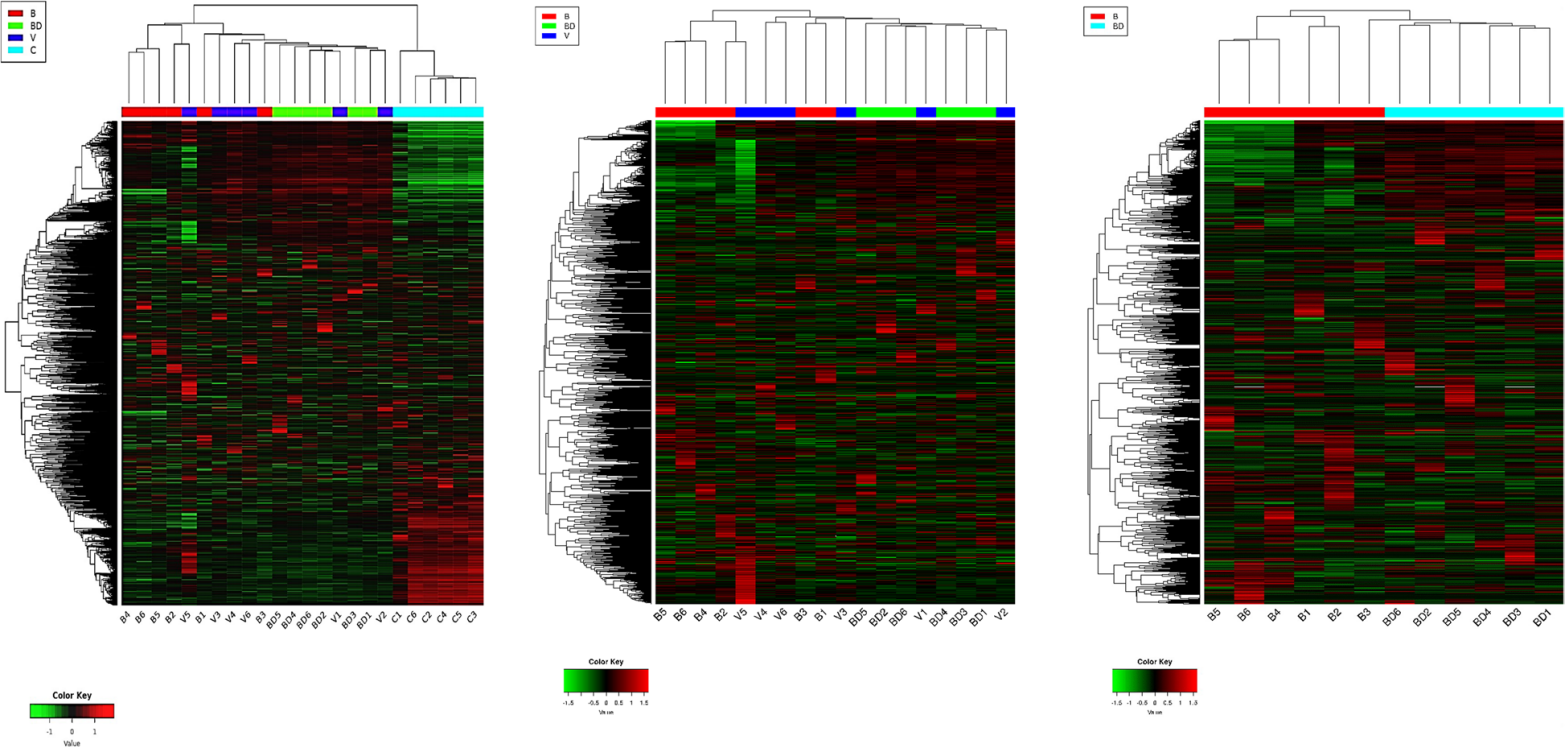
Expression signature of human A549 lung cancer cells cultured in sera from deer mice differing in bonding history. Unsupervised hierarchical clustering of whole transcriptome RNAseq data. In the graph on the left, sera from B, BD, and V were included, as well as FBS (designated as C). C was excluded in the middle graph, while in the right graph, Both C and V were excluded. FPKM values were used, and the 2,000 most variable genes were considered

A549 human lung cancer cells were cultured in the presence of sera from male *P. californicus* that differed in their bonding histories as described in ref. 16. Those included virgin (V), bonded (B), and bond-disrupted (BD) individuals. Cells cultured in FBS were also included as controls (C). RNA sequencing was performed, and the whole transcriptomes were subjected to unsupervised hierarchical clustering analyses. The results indicated that the transcriptomes clustered relatively close together, based on the bonding experience of the original serum donors (Fig. 2). The species from which the serum was derived was the most potent discriminator of expression profiles. The cells that grew in *Peromyscus* serum were accurately grouped together, and the same was observed for the cells grown in FBS (Fig. 2). Thus, species-specific factors can differentially modulate the transcriptome of lung cancer cells, instructing them to acquire distinct expression profiles. Among the Peromyscus sera-derived samples, those corresponding to the bonded and the bond-disrupted animals also clustered well together (Fig. 2). Samples corresponding to the virgin group exhibited the lowest stringency in unsupervised clustering since some individual animals clustered better with the bonded while others with the bond-disrupted groups. An intriguing possibility is that this observation indicates the lowest rigidity in the expression profile induced by virgin animals' sera, as opposed to the more robust transcriptomic changes triggered by the sera of bonded and bond-disrupted animals. Nevertheless, when whole lungs were analyzed instead of cells cultured in deer mouse sera, virgins were effectively discriminated (Fig. 1a). Specific gene transcripts in differential gene expression analysis have been described elsewhere [16].

Genetic relatedness did not appear to be of significance (Fig. 2). Some of the serum samples used for the bonded and bond-disrupted animals corresponded to the same individuals, with serum samples obtained at bonding and subsequently at bond disruption (B1, B2, and B3 correspond to BD2, BD5, and BD6, respectively). These specimens did not show similarities that could surpass those directed by the bonding experiences. Furthermore, V1 and V5 were siblings of B6 and BD3, respectively but clustered distally from them. Thus, clustering is directed primarily by the species, followed by the social experience, while genetic relatedness does not appear to be of importance.

### Expression signature of pair bonding and disruption

**Fig. 3:**
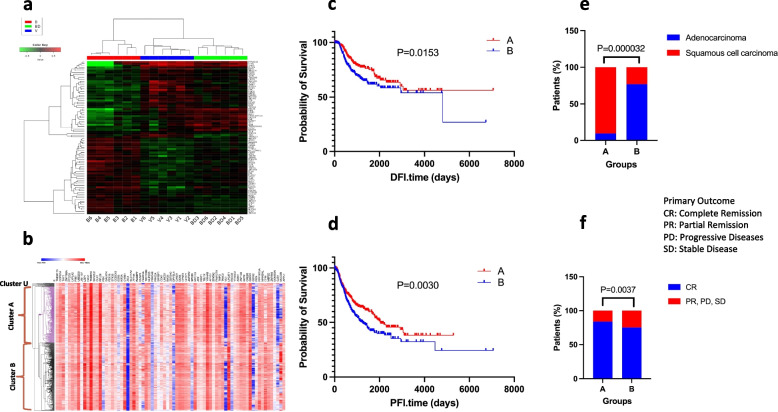
Bonding signature and lung cancer prognosis. **a**. The expression signature of 88 genes in A549 human lung cancers is sufficient to predict the bonding history of serum donors. **b**. Heatmap of expression data in patients with primary lung cancer (TCGA dataset) for 75 out of 86 genes described in (a). Unsupervised hierarchical clustering discriminates two major clusters (indicated as A and B) and a third minor one (cluster U). KM plots for the patients of clusters A and B, as described in (b), exhibited significant differences in disease-free interval time (c) *P* = 0. 0153; Log-Rank, Mantel-Cox test), and progression-free interval (**d**) *P* = 0.0030; Log-Rank, Mantel-Cox test). Bar plots showing the primary diseases (**e**) *P* = 0.000032 chi-squared test) and primary outcome (**f**) *P* = 0.0037 chi-squared test) of the patients of clusters A and B, as described in (b). CR: Complete Remission; PR: Partial Remission; PD: Progressive Disease; SD: Stable Disease

The observed tendency of expression profiles to cluster together in relation to the bonding history of the original serum donors prompted us to investigate whether an expression signature can be defined that could be used to accurately discriminate the cultured lung cancer cells according to the social experiences of the original serum donors [19]. To that end, initially, we identified the transcripts that are differentially expressed in each group as compared to all others (*P* < 0.05 in unpaired t-tests in all pairwise comparisons between the RNAseq data). This analysis produced a roster of 88 transcripts that accurately predicted the bonding experience of the serum donors (Fig. 3a). Clustering by using this 88-gene signature divided transcriptome data into two branches, consisting of the samples from the bonded group and the bond-disrupted and virgin groups combined (Fig. 3a). The fact that virgins and bond-disrupted animals exhibited the lowest degree of discrimination, likely reflects the fact that at bonding, serum inflicts a more robust expression profile, while the profiles associated with virgin and bond-disrupted animals have higher similarities between them than each has with the bonded group.

Noteworthy, virgin serum donors were group-housed while bond-disrupted animals were housed alone for at least 1 week after the separation from their partners. This is ruling out the possibility that the similarity of virgins and bond-disrupted animals is due to social interactions, a potential consequence of the fact that *P. californicus* is particularly sensitive to social stress [20–22] and further supports the notion that bonding of mating partners is associated with unique changes that are distinct and prevail from those of social interactions per se [23, 24].

### Bonding signature, lung cancer prognosis and p53 mutations

**Fig. 4:**
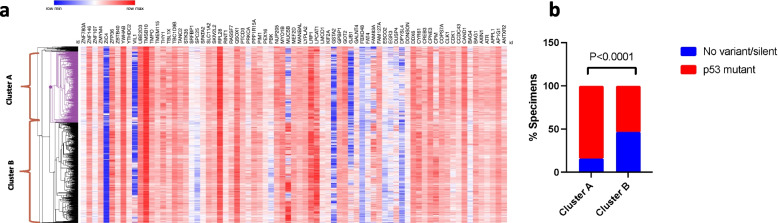
Bonding signature and p53 mutations. a. Heatmap of gene expression data in patients with primary lung cancer (TCGA dataset) for the 75 out of 86 genes described in (Fig. 3a). b. Unsupervised hierarchical clustering discriminates two major clusters (indicated as A and B) that differed in the prevalence of p53 mutations (*P* < 0.0001, chi-squared test)

To explore if the 88-gene signature described above carries clinical value, we tested it in lung cancer cases from the publicly available TCGA database (https://xenabrowser.net/). We retrieved expression data for 75 out of 88 genes and analyzed them using unsupervised hierarchical clustering. As shown in Fig. 3b, the application of this 75-genes signature discriminated the human tumor specimens into two major clusters (cluster A and B) and a third, smaller unassigned cluster (cluster U). It is likely that the unavailability of data for 13 genes of the original 88-gene signature contributed to the un-assignment of cluster U. Furthermore, the two clusters A and B exhibited a significant difference in disease-free interval time (Fig. 3c), progression-free interval (Fig. 3d), primary diseases (Fig. 3e), and primary outcomes (Fig. 3f) implying clinical relevance. For primary disease, the TCGA database includes only information on two subtypes of non-small cell lung cancer (adenocarcinoma and squamous cell carcinoma). Cluster A has a higher probability of survival than cluster B considering both parameters (DFI.time and PFI.time). As indicated by graph 3e, the proportion of people diagnosed with primary squamous cell carcinoma in cluster A is higher than that in cluster B, and there is a greater proportion of complete remissions to partial remissions, stable disease, and progressive disease in cluster A than in cluster B (Fig. 3f). The value of this 75-gene signature also extends to the molecular profile of the tumors. p53 mutations are common in lung cancer and are associated with clinical outcomes [25]. Mutation data in p53 was available in 952 specimens of those used for the analysis of the 75-gene signatures. Application of this signature discriminated the lung tumors into two major groups (Cluster A and B in Fig. 4a) that significantly differed in p53 status: In cluster A, 84% of the tumors harbored genomic alterations in p53 as compared to only 53% in cluster B (*P* < 0.0001, chi-squared test) (Fig. 4b).

### Proteomic analysis of sera from bonded and bond-disrupted animals

**Fig. 5:**
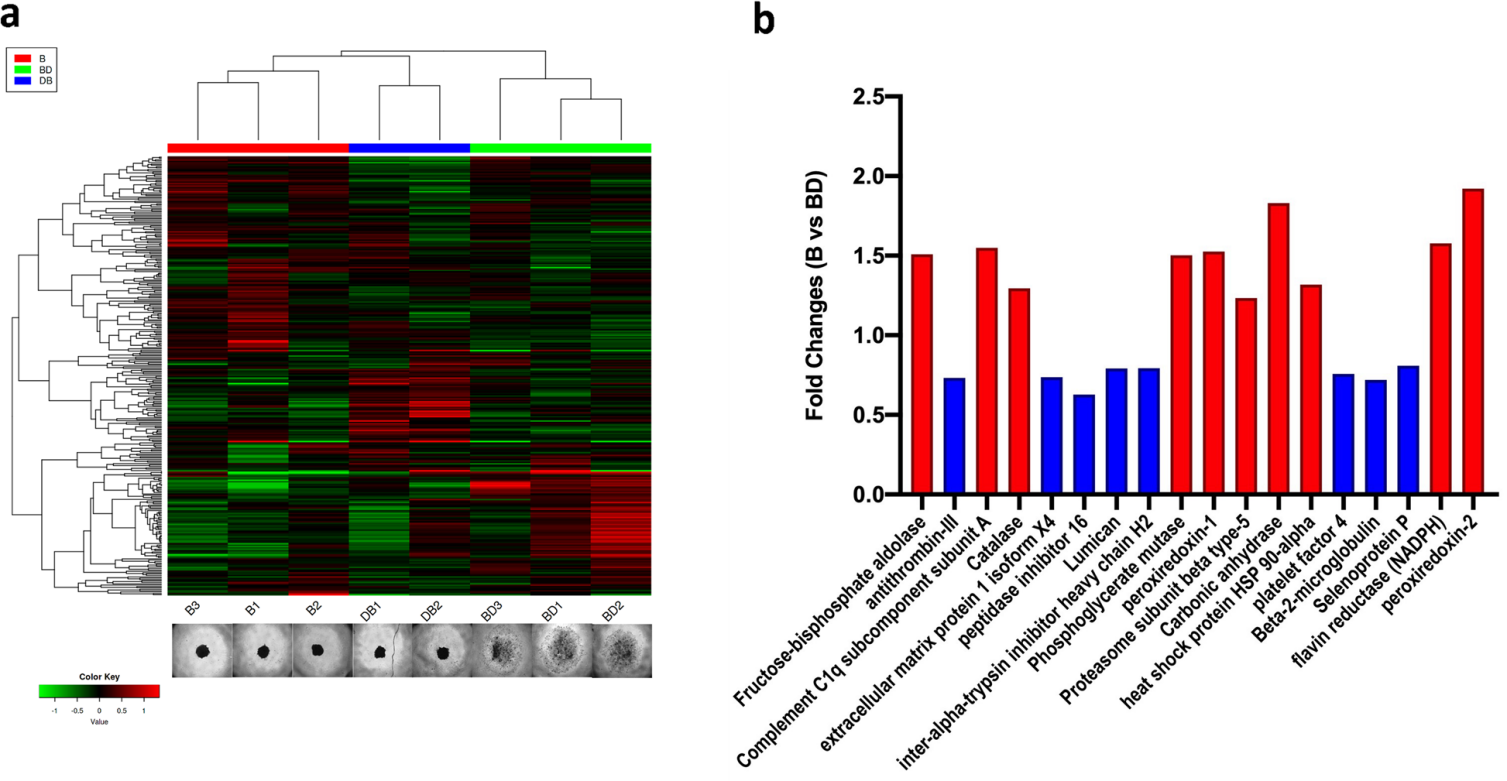
Proteomic studies. **a**. Unsupervised hierarchical clustering of 318 proteins’ abundance that was detected in the sera of bonded (B) and bond-disrupted (BD and DB) animals. The two groups of samples from bond-disrupted animals reflected the effects of the sera in spheroid formation (shown at the bottom; ref. 16). In DB1 and DB2 a notable effect in spheroid size was not detected, as opposed to BD1-3. **b.** Protein expression levels exhibited a significant difference between the bonded and bond-disrupted groups. Red bars indicate upregulated and blue bars downregulated proteins (*P* < 0.05; student’s t-test)

To further explore the basis for the differential effects of deer mouse sera, samples isolated from bonded (*n* = 3) and bond-disrupted animals (*n* = 5) were subjected to mass spectrometry-based proteomic analysis. A total of 318 proteins were identified. Protein abundance clustered the samples together according to bonding history (Fig. 5a). Bond-disrupted specimens formed two groups that reflected the effects of sera in A549 microsphere formation (as described in ref. 16). The sera samples that had only minimal effects in spheroid size (designated as DB in Fig. 5a) segregated closer to the bonded samples while the ones that considerably promoted spheroid formation (designated as BD in Fig. 5a) segregated more distally. Two sibling pairs were also included, in B and BD, and in B and DB, but similarly to the RNAseq analysis, clustering followed bonding experiences. Differential protein expression revealed 18 proteins that had significant differences in levels between the bonded and bond-disrupted groups (Fig. 5b). Pathway enrichment could not reliably be performed because of the small number of differentially expressed proteins. Nevertheless, several of these proteins were associated with the metabolism of reactive oxygen species and the regulation of redox equilibria (Catalase, peroxiredoxin-1, heat shock protein HSP 90-alpha, flavin reductase (NADPH), peroxiredoxin-2), and all these proteins have a higher expression rate in the bond-disrupted group (red color).

## Discussion

While both the epidemiological and the anecdotal and experiential evidence clearly point to the effects of social interactions in physiological processes, their mechanistic basis remains poorly defined. Furthermore, it is also doubted the extent by which such effects possess a physiological foundation or if they primarily reflect lifestyle changes. We demonstrate using monogamous deer mice that social interactions, in particular those that occur on the basis of mating, affect peripheral tissues such as the lungs by influencing their transcriptomic profiles. More importantly, the changes occur in a coordinated manner that effectively results in the discrimination of the specimens according to the bonding history and current bonding status of the animals. It is noteworthy that such changes masked the potential effects of genetic relevance because animals clustered with other animals that had similar bonding experiences rather than their siblings with whom they share a genetic heritage. It is noted that in the absence of siblings, at some instances, cousins were used which represents a limitation of the study which suggests that the interpretation must be considered with some caution. Furthermore, the enriched pathways involve, among others, the metabolism of oxygen and the development of the heart, underscoring the relationship between bonding status and lung function. It is plausible, and indeed experimentally testable, that such alterations reflect physiological adaptations related to heart beating and breathing rates that may occur during bonding and are modulated at disruption.

Analogous changes to those detected in the lungs of animals were also seen in blood sera and were found equally potent in discriminating lung cancer cells according to the bonding history of serum donors. For example, peroxiredoxins 1 and 2 were significantly upregulated in the serum proteomic analysis, and Prdx6 encoding for peroxiredoxin 6, was the most highly upregulated gene in the lung RNAseq analysis. Furthermore, evidence for the impact of changes in pathology was derived by the observation that a 75-gene signature that effectively discriminated cancer cells also predicted disease outcomes in primary lung cancer patients, as well as in p53 mutations that influence prognosis. It seems plausible that bonding history alters the abundance of certain soluble factors in circulation, which in turn can elicit specific changes in the tumors. These changes collectively define expression signatures that are associated with lung cancer prognosis and therapeutic outcomes in human patients. As indicated by proteomic studies, such factors may involve, at least partially, proteins implicated in the regulation of redox status. Thus, in addition to the direct effects of the hormones that regulate pair bonding, physiological changes associated with oxygen metabolism may also contribute to the adverse effects of bond disruption.

Interestingly, while bonded and bond-disrupted specimens were accurately discriminated in both lungs and cancer cells cultured in deer mouse sera, specimens from virgin animals were only discriminated in lung tissue analysis. In sera-cultured cancer cells, virgins either clustered with bonded, bond-disrupted (whole transcriptome), or with the bond disrupted (75-gene signature). This is consistent with the lower robustness of the virgin-induced signature, which is more dependent on the specific roster of transcripts used to describe it. It is of note, however, that in the experiment that involved serum, FBS elicited the most distinct profile. This may raise concerns regarding the application of cell culture findings involving FBS in physiologically relevant conditions.

Based on these findings, it is likely that various types of social interactions induce distinct expression signatures in the periphery, altering their physiology and, consequently, modulating disease susceptibility [26, 27]. Consistently with these notions, in voles, disruption of pair bonds has been linked extensively to the deregulation of oxytocin signaling, a neurohormone that produces diverse effects in peripheral tissues in both physiological conditions and in tumors [28–32]. Differential changes dependent on mating status have also been described in different brain regions of monogamous and polygamous voles, which may alter, directly or indirectly, the abundance of various soluble factors in the sera [33–36]. Furthermore, in mice, social isolation inflicts changes that are reflected in the expression profiles of peripheral tissues and influence tumorigenesis [37–39], while in rats, hamsters, and Peromyscus affects the efficacy of wound healing [40–43].

Collectively, these results imply that the individuals’ social experiences, such as bond formation, modify the transcriptome of the lungs and modulate disease prognosis. To that end, bonding history emerges as a potent modifier of physiological and pathological processes and should be considered when therapeutic options are evaluated. Finally, the coordination of the lung, and potentially of other tissues’ transcriptomes with respect to the bonding status of the individuals provide an additional physiological foundation of connectedness, implying similarities in biobehavioral characteristics and disease predisposition [44].

## Materials and methods

### Animals

Genetically diverse male *P. californicus* (stock IS), 12–15 months old, were obtained from the Peromyscus Genetic Stock Center (Columbia, SC) (RRID:SCR_002769). Animals were divided into three experimental groups, including bonded, bond-disrupted, and virgins. For the bonded and bond-disrupted mice, we used individuals that were paired for about 4 months. For bond-disrupted animals, mice were separated after four months, and lung tissues were collected one week later. For virgin mice, lung tissues were collected from mice of similar age housed in groups of three in each cage. Animal studies were approved by the University of South Carolina IACUC (Protocol # 2473–101,464-102319).

### RNA analysis

RNAseq analysis of A549 cells was reported in ref. 16. A549 cells were originally obtained by ATCC. For tissue analysis, RNA was extracted using the Qiagen Rneasy Mini Kit (Qiagen, 74,106) according to the manufacturer’s instructions. Dnase was added to remove contaminating genomic DNA using the Rnase-Free Dnase Set (Qiagen, 79,254). RNA was eluted into 250 ng/µl of nuclease-free water and sent for RNA integrity assay and RNA sequencing as described [16]. Hierarchical clustering analysis was performed using the Morpheus analysis software (https://software.broadinstitute.org/morpheus) for the human lung cancer data or iDEP1.12 for the analysis of the Peromyscus data [45]. For the analysis of the Peromyscus data, FPKM values were used. A FDR cutoff of 0.1 and fold change ≥ 2 were considered in the DEG analysis of lung RNA sequencing data. Analysis for differential gene expression was performed by using the limma package as integrated in iDEP1.12 and enrichment analysis was performed using the north american deer mouse lung RNA [46].

### Proteomic analysis

For the bonded group, sera were collected from animals aged 19 to 21 months who had been paired for 13 months, whereas for the bond-disrupted groups, sera were collected from animals with similar pairing times and ages one week after the bond disruption. The analysis was performed by Creative Proteomics (Shirley, NY) by mass spectrometry of TMT-labeled samples. The full scan was performed between 350–1,650 m/z at the resolution 120,000 at 200 Th. Protein IDs were assigned following alignment to the Peromyscus maniculatus protein database.

Statistical analysis. Statistical analysis was performed by unpaired t-test, chi-squared test, or Log-rank (Mantel-Cox) test as indicated in the figure legends and text. Results were considered significant when P ≤ 0.05. Graphs were generated using GraphPad Prism software (version 8).

### Supplementary Information

**Additional file 1:  Table 1. **Supplementary to Figure 1b. Differentially expressed transcripts in the lungs of virgin (V), bonded (B) and bond-disrupted (BD) male P. californicus.

**Additional file 2: Table 2. **Supplementary to Figure 1b. Enrichment pathway for differentially expreessed gene between BD vs V.

**Additional file 3: Table 3. **Supplementary to Figure 1c. Enrichment of pathways in cluster C.

## Data Availability

All data and materials are available upon request. Transcriptomic data have been deposited to NCBI Gene Expression Omnibus ID GSE167827 and GSE229537.
